# SARS-CoV-2 Pathogenesis: Imbalance in the Renin-Angiotensin System Favors Lung Fibrosis

**DOI:** 10.3389/fcimb.2020.00340

**Published:** 2020-06-12

**Authors:** M. Victoria Delpino, Jorge Quarleri

**Affiliations:** ^1^Facultad de Farmacia y Bioquímica, Instituto de Inmunología, Genética y Metabolismo (INIGEM), Universidad de Buenos Aires, Buenos Aires, Argentina; ^2^Consejo Nacional de Investigaciones Científicas y Técnicas (CONICET), Buenos Aires, Argentina; ^3^Facultad de Medicina, Instituto de Investigaciones Biomédicas en Retrovirus y Sida (INBIRS), Universidad de Buenos Aires (UBA), Buenos Aires, Argentina

**Keywords:** SARS-CoV-2, ACE2, Fibrosis, ANG1-7, Renin angiotensin system (RAS)

## Introduction

After SARS-CoV-2 infection, a major complication of those who survived to COVID-19 outbreak is the development of severe lung disease leading to pulmonary fibrosis. At earliest step of virus-host cell interaction when the SARS-CoV-2 interacts with the ACE2 receptor highly expressed in pneumocytes type II, a linkage is established between the renin-angiotensin-system (RAS) and the viral pathogenesis. Within this important system, the angiotensin-converting enzyme (ACE) is deputed to the conversion of angiotensin I to angiotensin II (AngII), a potent vasoconstrictive peptide involved directly in inflammation and fibrosis development. AngII is hydrolyzed by ACE2 to Ang1-7, triggering a cascade of events that counteract fibrosis. This imbalance is known to be due to inflammatory damage. However, because ACE2 is the receptor for SARS-Cov-2, we could also speculate that the virus *per se* could modulate its enzymatic activity. In our opinion the wound healing pathways that mediate tissue repair after SARS-CoV-2 mediated injury, should consider managing the imbalanced ACE/ACE2 axis. We hypothesize that the heptapeptide Ang1-7 could provide novel therapeutic interventions for pulmonary fibrosis patients. Understanding how the RAS, wound healing and other pro-fibrotic pathways act after viral infection should lead to novel therapeutics in the future.

## Development of Lung Fibrosis and SARS-Cov-2

In humans, there is an extensive information currently available supporting a clear correlation between the development of pulmonary fibrosis and respiratory viral infections (Sheng et al., 2019). The lung architecture and function are altered by the progressive enlargement of fibroblasts population and extracellular matrix. Enhanced attention has been directed to airway remodeling (Holgate, 2011). There both TGF-β1 (transforming growth factor-β1) and collagen may play critical roles in the formation of airway remodeling. However, the underlying molecular mechanisms that occurs once viral infection is established leading to fibrosis remain obscure until present.

To date, based on both the observation of the clinically defined as severe cases of the Coronavirus Disease 2019 (COVID-19) caused by SARS-CoV-2 (Severe Acute Respiratory Syndrome CoV-2), as well as the analysis of biopsy/autopsy materials (presence of inflammatory clusters with fibrinoid material and multinucleated giant cells, with interstitial fibroblasts), it is permeable to establish some similarities with findings reminiscent of the SARS-CoV, responsible for the severe respiratory distress syndrome (SARS) that emerged in 2002–2003 (Huang et al., 2020; Schaller et al., 2020; Tian et al., 2020). Comparison of amino acid sequences revealed a high similarity (95–100%) between most of the SARS-CoV-2 proteins and those of SARS-CoV (Grifoni et al., 2020). During the acute phase of SARS-CoV infection, lung damage causes edema, alveolar shedding of epithelial cells, and the deposition of hyaline material in the alveolar membranes, reducing the efficiency for gas exchange. During the next phase of infection (weeks 2–5), the lungs show signs of fibrosis, noting the deposition of fibrin and infiltration of inflammatory cells and fibroblasts close to the epithelial cells, in the alveolar spaces. During the final stage (weeks 6–8), the lung tissue becomes fibrotic with collagen deposits, and epithelial cell proliferation is observed in alveoli and interstitial spaces (Ye et al., 2007). The available evidence on the pathological processes associated with SARS-CoV involves both direct cytopathic effects on epithelial cells, as well as aberrant activation of the innate immune response. Thus, this virus is capable of promoting the activation of intracellular stress promoting pathways, lysosomal damage and the consequent activation of autophagy, to preserve cell viability. In this multifactorial context, autophagy, and oxidative stress merit attention. Recognized as a dynamic and complex regulatory process, autophagy may play a central role in pulmonary fibrosis, depending on the cell type and condition against infection. Thus, under normal conditions in alveolar epithelial cells (type I- and II-pneumocytes), alveolar macrophages and endothelial cells, autophagy could be activated to maintain its homeostasis, inhibit its death, and prevent fibrosis development (Zhao et al., 2020).

From the first histopathological descriptions, the molecular basis of the pulmonary fibrosis progression due to SARS-CoV-2 infection is still unclear, and could be complex and multifactorial, involving direct viral effects, immune dysregulation/cytokines (MCP-1; IL-6, IL-8, TGF-β, TNF-α), and increased oxidative stress (Liu J. et al., 2020; Xu et al., 2020).

Some insights into the mechanisms leading to COVID-19 associated fibrotic process could be shared with those associated with chronic idiopathic pulmonary fibrosis. Therefore, even without addressing the immune dysregulation of SARS-CoV-2 infection, in spite of beneficial effects, the available antifibrotic therapy could exacerbate other clinical aspects of the infection such as the liver and renal pathology (George et al., 2020).

## The Renin–Angiotensin System (RAS) in Lung Homeostasis and Pathogenesis

The renin–angiotensin system (RAS) is an endocrine system involved in cardiovascular regulation, and water balance. The RAS carries on biological functions that are modulated by a series of stimuli to preserve physiological hemostasis. The pathogenesis of hypertension, myocardial infarction, heart failure, diabetes, and inflammatory lung disease pathogenesis involves an abnormal RAS activation (Jia, 2016). Besides, the airway remodeling depicted by patients with exacerbated lung fibrosis, has been associated with elevated plasma levels of AngII (angiotensin II), which could trigger TGF-β1 production and collagen deposition (Uhal et al., 2007; Gao et al., 2009; Yang et al., 2009). In the RAS, the ACE (angiotensin-converting enzyme)–AngII–AT1 (AngII receptor type 1) axis activation causes deleterious effects, including vasoconstriction, inflammation, and fibrosis (McKay et al., 1998). The AngII is hydrolyzed by the enzyme ACE2, generating the angiotensin heptapeptide Ang1-7 able to interact with its specific Mas receptor. This alternative ACE2–Ang1–7–Mas axis appears to counter-regulate the ACE–AngII–AT1 axis (Santos et al., 2013). In this context, Ang1–7 has been shown to have anti-thrombotic, anti-proliferative, anti-fibrotic, and anti-inflammatory properties in heart, kidney, and arthritis animal model (Gava et al., 2009; da Silveira et al., 2010). Furthermore, a vast range of advantageous effects of Ang1-7 or its analogs with a longer half-life has been documented, mainly through Mas receptor interaction, exerted on different anatomic locations and tissues (Passos-Silva et al., 2013; Machado-Silva et al., 2016).

In addition to its functions in regulating blood pressure, AngII plays a pivotal role in signaling cellular and molecular events that are considered critical in the pathogenesis of pulmonary fibrosis, such as: (i) inflammation (promoting production of proinflammatory cytokines such as IL-6, and IL-8 by macrophages), (ii) the production of reactive oxygen species (ROS) among infected-alveolar epithelial cells followed by its apoptosis, and (iii) the proliferation, migration, and differentiation of fibroblasts to myofibroblasts capable of synthesize smooth muscle alpha-actin (α-SMA) and produce extracellular matrix (collagen and fibronectin) through a mechanism mediated by autocratic trans-activation of TGF-β in the fibroblast itself (Wolf et al., 1992; Kagami et al., 1994; Jia, 2016). In contrast, the Ang1-7 peptide, after interacting with its cellular receptor Mas, exhibits the ability to inhibit proapoptotic signaling in alveolar epithelial cells, promote autophagy, and—together with the ACE2 receptor—counteract the profibrotic effects, reducing both TGF-β mediated collagen expression, as well as the transition from fibroblasts to myofibroblasts (Iwata et al., 2005; Zeng et al., 2009; Zhou et al., 2016).

## SARS-Cov-2, RAS, and Lung Fibrosis

The direct virus-host interaction begins with the adsorption step in the viral replication cycle. Here, it involves the high affinity binding between the viral spike (S) protein with the ACE2, followed by the S cleavage by the cellular transmembrane protease serine 2 (TMPRSS2) action, thus favoring the virus entry (Zhou et al., 2020). In normal conditions, the ACE2 is widely expressed near the surface of various epithelial cells—blood vessels, lung, intestine, and others. Although, during lung fibrosis, such expression by a c-Jun N-terminal kinase (JNK)-mediated transcriptional pathway, is downregulated depending on the cell-cycle stage. In the adult lung, the major sources of angiotensin-converting enzyme (ACE)-2 are the normally quiescent alveolar epithelial type II pneumocytes, that, during lung fibrosis, proliferate actively, and downregulate the expression of this protective enzyme. The ACE2 expression is severely downregulated or absent in actively proliferating pneumocytes during lung fibrosis (type I-pneumocytes), that appear replacing the damaged alveolar type II pneumocytes (Uhal et al., 2013). Moreover, a deregulation of this lung protective pathway may occur when the expression level of ACE2 is diminished after the interaction with the coronavirus SARS-CoV by its internalization inside the cell or, alternatively when it is released by TACE (ADAM17)-mediated cleavage from the surface of epithelia to the extracellular environment into the airway surface liquid (Kuba et al., 2005; Lambert et al., 2005; Jia et al., 2009). From these findings, it is plausible that ACE2 activity and the ACE2–Ang1–7–Mas axis are diminished after its binding to SARS-CoV-2. It may enhance the ACE-AngII–AT1 axis thus heightened AngII activity leading to pulmonary vasoconstriction and inflammatory and oxidative organ damage, increasing the acute lung injury risk. Supporting these assumptions, significantly higher serum AngII levels accompanied by higher viral load in respiratory secretions and severe lung injury among patients with COVID-19 pneumonia in comparison with healthy individuals (Liu Y. et al., 2020). The respiratory distress presented by severe SARS-CoV-2 infections is an unfavorable sign that could be directly related to the level of fibrosis and inflammation, favored by a cytokine storm involving IL-6, IL-10, and TNF-α (Chen et al., 2020).

When repetitive cycles of productive SARS-CoV infection occur in type II pneumocytes (epithelial cells in a quiescent state with high ACE2 expression) followed by cytolytic effect, their differentiation toward proliferating pneumocytes (low expression of ACE2) is promoted (Sims et al., 2008). TGF-β is a pivotal protagonist highly expressed in almost all fibrotic processes acting as potent pro-fibrogenic cytokine. Besides the well-recognized Smad-dependent cascade in TGF-β signaling, there is cumulative evidence indicating that ROS level also modulates such signaling through Smad-independent pathways. TGF-β and ROS are involved in a vicious cycle. On the one hand, TGF-β favors a redox imbalance by increasing ROS level and suppressing antioxidant enzymes. Besides, ROS induces TGF-β thus promoting its fibrogenic consequences (Liu and Desai, 2015). Interestingly, the heptapeptide Ang1-7 is able to interfere with this pathway by diminishing the AngII-elicited expression of Smad proteins and the nuclear trafficking of p-Smad2/3, as well as by decreasing the level of phosphorylation of PI3K (phosphoinositide 3-kinase), Akt, p38-MAPK (mitogen-activated protein kinase), and JNK (c-Jun N-terminal kinase) signaling pathways (Zhou et al., 2016).

**Figure 1:**
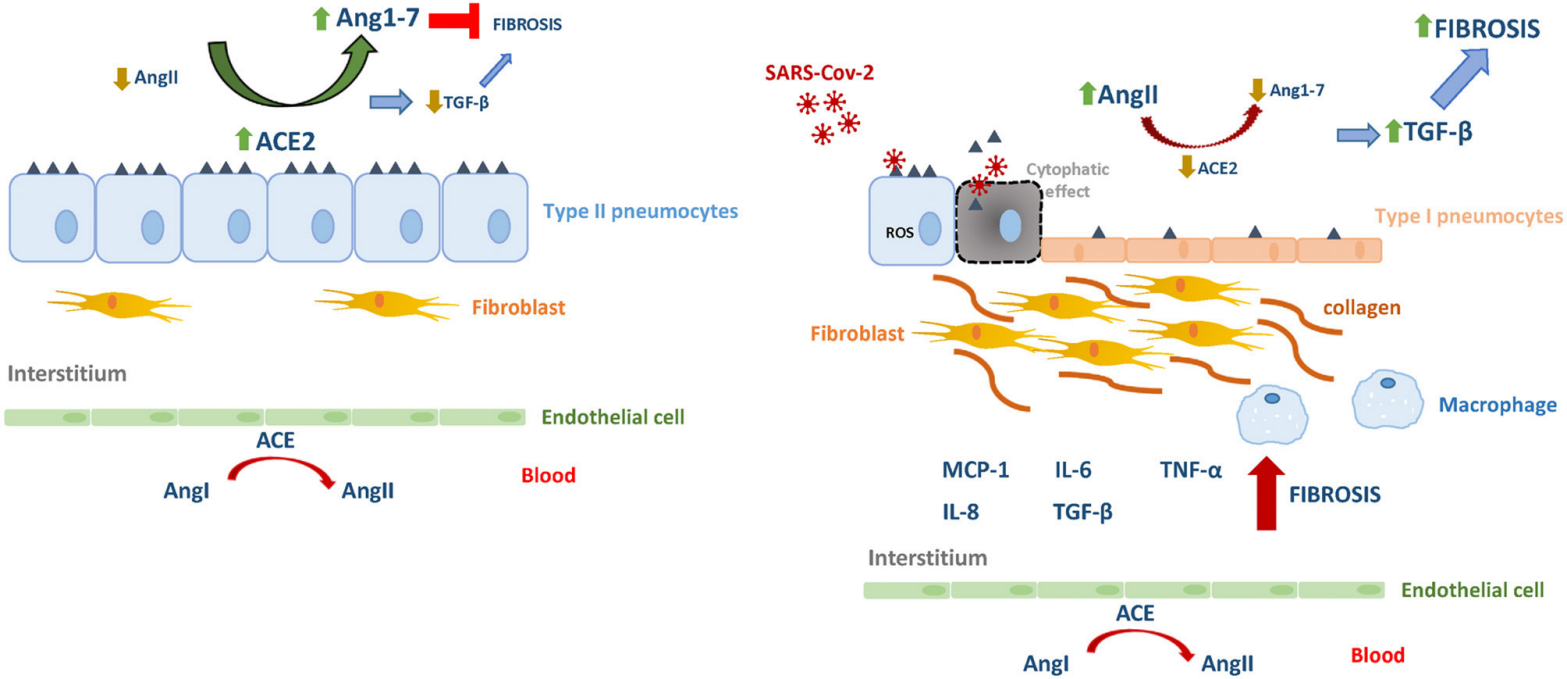
The renin–angiotensin system in homeostasis and in SARS-Cov-2 infection. Angiotensin I (AgnI), Angiotensin II (AgnII), Angiotensin 1-7 (Agn1-7), angiotensin-converting enzyme (ACE), angiotensin-converting enzyme 2 (ACE2).

In pulmonary viral infection-induced fibrosis, the oxidative stress rises in epithelial cells, thus stimulating the production and release of TGF-β, causing excessive migration, proliferation, activation, and myofibroblastic differentiation of fibroblasts, causing the abnormal accumulation of these cells and reflecting the process of airway remodeling. Myofibroblasts are a major producer of collagenous and non-collagenous matrix molecules and its hyperplasia has been demonstrated in asthmatic patients (Yang et al., 2012; Sakai and Tager, 2013). On the other hand, AngII-induced collagen expression also depends on TGF-β (Kagami et al., 1994), which subsequently induces extracellular matrix accumulation and inflammation. In this scenario, activated fibroblasts induce further injury and death of alveolar epithelial cells, thereby creating a vicious circle of profibrotic epithelial cell-fibroblast interactions nourished by TGF-β leading to the formation of non-functional scar tissue (Li et al., 2016). Also, TGF-β would also be responsible for the inhibition of the expression of the Mas receptor for Ang1-7 in fibroblasts, thereby antagonizing the anti-fibrotic capacities of the hepatapeptide (Cofre et al., 2015). In this microenvironment, TGF-β will be able to act on alveolar macrophages stimulating the secretion of IL-4, IL-6, and IL-13, thus enhancing the development of fibrosis. As a counterpart, inhibition of TGF-β is expected to decrease the influx of neutrophils, macrophages, and lymphocytes at the site of injury. In contrast, Ang1–7 could inhibit AngII-induced expression of TGF-β, α-SMA and collagen, as it was demonstrated at different tissues (Zeng et al., 2009; Shenoy et al., 2010; Marques et al., 2012) (Figure 1).

## Concluding Remarks

In conclusion, early events during the SARS-CoVs infection propitiate the imbalance the RAS favoring increased levels of AngII, thus promoting inflammation, and exacerbated fibrosis. The current knowledge offers the chance to counteract such cascade of pathogenic events by increasing Ang1–7, able to inhibit TGF-β and collagen expression, contributing to a potential attenuation of airway remodeling during severe COVID-19.

## Author Contributions

MD and JQ conceived the idea and drafted the manuscript. All authors contributed to the article and approved the submitted version.

## Conflict of Interest

The authors declare that the research was conducted in the absence of any commercial or financial relationships that could be construed as a potential conflict of interest.
